# Significance of sTREM-1 in early prediction of ventilator-associated pneumonia in neonates: a single-center, prospective, observational study

**DOI:** 10.1186/s12879-020-05196-z

**Published:** 2020-07-25

**Authors:** Xingxing Zhao, Lixiao Xu, Zuming Yang, Bin Sun, Ying Wang, Gen Li, Chenxi Feng, Tao Pan, Tian Yu, Xing Feng

**Affiliations:** 1 Department of Neonatology, Suzhou Municipal Hospital, The Affiliated Suzhou Hospital of Nanjing Medical University, Suzhou, 215000 China; 2 Institute of Pediatric Research, Children’s Hospital of Soochow University, Suzhou, 215000 China; 3 Department of Neonatology, Children’s Hospital of Soochow University, Suzhou, 215000 China

**Keywords:** Neonate, Mechanical ventilation, Ventilator-associated pneumonia, Biomarker, Soluble myeloid cell trigger receptor-1

## Abstract

**Background:**

To evaluate whether soluble triggering receptor expressed on myeloid cells-1 (sTREM-1) can be used as an early predictor of ventilator-associated pneumonia (VAP).

**Methods:**

Ventilated neonatal patients admitted into the neonatology department between January 2017 and January 2018 were divided into VAP (*n* = 30) and non-VAP (*n* = 30) groups. Serum sTREM, procalcitonin (PCT), C-reactive protein and interleukin-6 levels were measured at 0, 24, 72, and 120 h after initiation of mechanical ventilation (MV). Correlations between blood biomarker concentrations and VAP occurrence were analyzed. Predictive factors for VAP were identified by logistic regression analysis and Hosmer-Lemeshow test, and the predictive value of sTREM-1 and biomarker combinations for VAP was determined by receiver operating characteristic curve analysis.

**Results:**

The serum sTREM-1 concentration was significantly higher in the VAP group than in the non-VAP group after 72 and 120 h of MV (72 h: 289.5 (179.6–427.0) vs 202.9 (154.8–279.6) pg/ml, *P* < 0.001; 120 h: 183.9 (119.8–232.1) vs 141.3 (99.8–179.1) pg/ml, *P* = 0.042). The area under the curve (AUC) for sTREM-1 at 72 h was 0.902 with a sensitivity of 90% and specificity of 77% for the optimal cut-off value of 165.05 pg/ml. Addition of PCT to sTERM-1 at 72 h further improved the predictive value, with this combination having an AUC of 0.971 (95% confidence interval: 0.938–1.000), sensitivity of 0.96, specificity of 0.88, and Youden index of 0.84.

**Conclusion:**

sTREM-1 is a reliable predictor of VAP in neonates, and combined measurement of serum levels of sTREM-1 and PCT after 72 h of MV provided the most accurate prediction of VAP in neonatal patients.

## Background

Ventilator-associated complications have increasingly drawn attention in ventilated neonatal patients due to their detrimental impact on prognosis. Ventilator-associated pneumonia (VAP) is one of the most commonly seen complications in neonatal patients [1], and it usually leads to a difficult ventilator weaning, which increases the duration of intensive care unit (ICU) stay [2]. The reported mortality rates among neonatal patients with VAP have been as high as 20–75% in both China and western countries [3–5]. Although early prediction of VAP could contribute to timely prevention and treatment, no effective predictors of VAP in neonates have been discovered so far [6]. The early diagnosis of VAP in neonates remains a challenge because clinical signs and radiological manifestations are non-specific, and tissue culture as the gold standard of diagnosis is time-consuming, invasive and easily contaminated.

Several blood biomarkers have been tested in previous studies for their ability to aid the diagnosis and prognosis of VAP. Inflammatory cytokines such as interleukin-6 (IL-6), C-reactive protein (CRP), and procalcitonin (PCT) have clinical significance as predictive or prognostic markers of infection [7–12]. Specifically, PCT was shown to be effective for guiding antibiotic treatment [13]. The sensitivity and specificity of PCT for predicting the prognosis of VAP were superior to those of CRP, but both values were found to be effective predictors of the response to antibiotic therapy [8, 14]. The serum IL-6 concentration was shown to be correlated with the severity of VAP but with limited predictive value based on the finding that IL-6 levels in serum and bronchoalveolar lavage fluid (BALF) did not differ significantly between patients with and without VAP [15]. Although studies of these biomarkers have provided important information regarding their value in VAP, the dynamic changes in the levels of these inflammatory biomarkers in neonates receiving mechanical ventilation (MV) have not been fully discovered and the possibility that a combination of these biomarkers could provide improved diagnostic or prognostic power in VAP has yet to be explored.

Myeloid cell trigger receptor-1 (TREM-1) is a member of the immunoglobulin super receptor family mainly expressed on the surface of myeloid cells such as neutrophils and monocytes/macrophages [16]. Soluble TREM-1 (sTREM-1), one of the two forms of TREM-1, has been reported as a novel and strong indicator of pneumonia [17]. To date, studies of the predictive value of sTREM-1 in pneumonia patients have mainly enrolled adult patients, and the possible significance of the serum concentration of sTREM-1 in neonates for the diagnosis and prognosis of VAP has not been investigated. Moreover, most studies have BALF samples to search for biomarkers for VAP. Importantly, sTREM-1 can be easily detected in blood samples, which are easier to obtain than BALF. The use of blood samples for measurement of biomarkers of VAP has been incompletely explored.

In this pilot study, we dynamically monitored the serum levels of sTREM-1 and the inflammatory cytokines PCT, CRP, and IL-6 in ventilated neonatal patients and investigated their diagnostic and predictive value for VAP.

## Methods

### Patients and study design

Patients admitted to the Neonatal Intensive Care Unit (NICU) at Children’s Hospital of Soochow University from January 2017 to January 2018 were prospectively screened. All patients who were diagnosed with VAP were included. The diagnosis was determined according to the criteria for VAP in infants under 1 year old published by the US Centers for Disease Control and Prevention (CDC) and the National Hospital Safety Monitoring Network (NHSN) [1]. Briefly, VAP was defined by the presence of at least three clinical manifestations including new or persistent infiltration changes occurring in the lungs and gas exchange disorders after 48 h of MV and within 48 h after weaning. Other inclusion criteria included a length of MV longer than 120 h and the absence of systemic infection following MV.

The exclusion criteria included systemic severe infection, incomplete medical records, MV duration less than 120 h, and surgical trauma.

All patients were ventilated with the MAQUET Servo-i ventilator system (Germany) or SLE 5000 Infant Ventilator (United Kingdom). Chest X-ray was performed immediately after MV, and routine X-ray was performed every 1–2 days thereafter.

The control group consisted of the same number of patients who received MV but did not develop VAP. The clinical characteristics of patients in the control group were well balanced with those of the VAP group in terms of gender, age, birth weight, gestational age, and disease severity.

### Sample collection and data recording

Baseline characteristics including name, gender, gestational age, birth day and other general conditions were recorded. Clinical signs including temperature, apnea, and nasal agitation were dynamically monitored along with laboratory and imaging results.

Peripheral blood samples were collected at the start of MV (0 h) and after 24, 72 and 120 h of MV. Blood samples were centrifuged at 2000 rpm for 5 min at room temperature, and the supernatant serum was collected and stored at − 80 °C. The serum concentrations of sTREM-1 and IL-6 were measured using enzyme-linked immunosorbent assay (ELISA) kits according to the manufacturer’s instructions (R&D Systems Inc., USA). CPR and PCT were determined at the local hospital laboratory. CRP test was based on the principle of the latex agglutination and PCT was determined by immunofluorescence staining.

### Statistical analysis

Normally distributed data were expressed as mean ± standard deviation (SD) and compared between groups using the independent sample t test. Non-normally distributed data were expressed as median with interquartile range and compared between groups using the Mann-Whitney U test. Categorical data were analyzed by the chi-square test or Fisher’s exact test. Correlations between two variables were calculated by the Spearman test. A general linear model was established for age correction. The Hosmer-Lemeshow test was performed to evaluate the goodness of fit for the logistic regression model. Receiver operating characteristic (ROC) curves were plotted and employed in combination with the results from logistic regression analysis to evaluate the efficacy of a single index and combined indexes for VAP prediction. SPSS version 22.0 (SPSS, Inc., USA) was used for statistical processing, and GraphPad Prism 5 (GraphPad, USA) was used for preparation of graphs.

## Results

### Patient characteristics

Thirty pediatric patients who received MV for at least 120 h and developed VAP as well as 30 matched patients who did not develop VAP were included in the study. From the 112 cases initially enrolled, we excluded 61 patients for whom the duration of MV was less than 120 h, 7 patients who had severe infection, 6 patients who had incomplete medical records and 8 patients who experienced surgical trauma. There were no significant differences in gender, mode of delivery, gestational age, birth weight, and underlying diseases between the VAP and non-VAP groups (Table 1).

**Table 1:** Baseline characteristics of neonatal patients in the VAP and non-VAP groups

Characteristics	VAP group (*n* = 30)	Non-VAP group (*n* = 30)	*P*
Gender (Male/Female)	20/10	21/9	0.780
Delivery mode (Natural/C-section)	11/19	9/21	0.580
Birth weight (g)	2403 ± 1073	2628 ± 912.4	0.384
Gestational age (weeks)	33.78 ± 3.807	35.64 ± 3.765	0.063
Age at admission (h)	8.5(3.75–27)	11.5(3.75–25)	0.830
Underlying diseases			
RDS (*n* = 22)	12	10	0.592
Malformation (*n* = 22)	10	12	0.592
Pneumothorax (*n* = 7)	4	3	1.000
Asphyxiation (*n* = 5)	2	3	1.000
Pulmonary hemorrhage (*n* = 3)	2	1	1.000
MAS (*n* = 1)	0	1	1.000

*Abbreviations*: *RDS* Respiratory distress syndrome, *MAS* Meconium aspiration syndrome

### Serum sTREM-1 concentration in neonatal patients with and without VAP

**Fig. 1:**
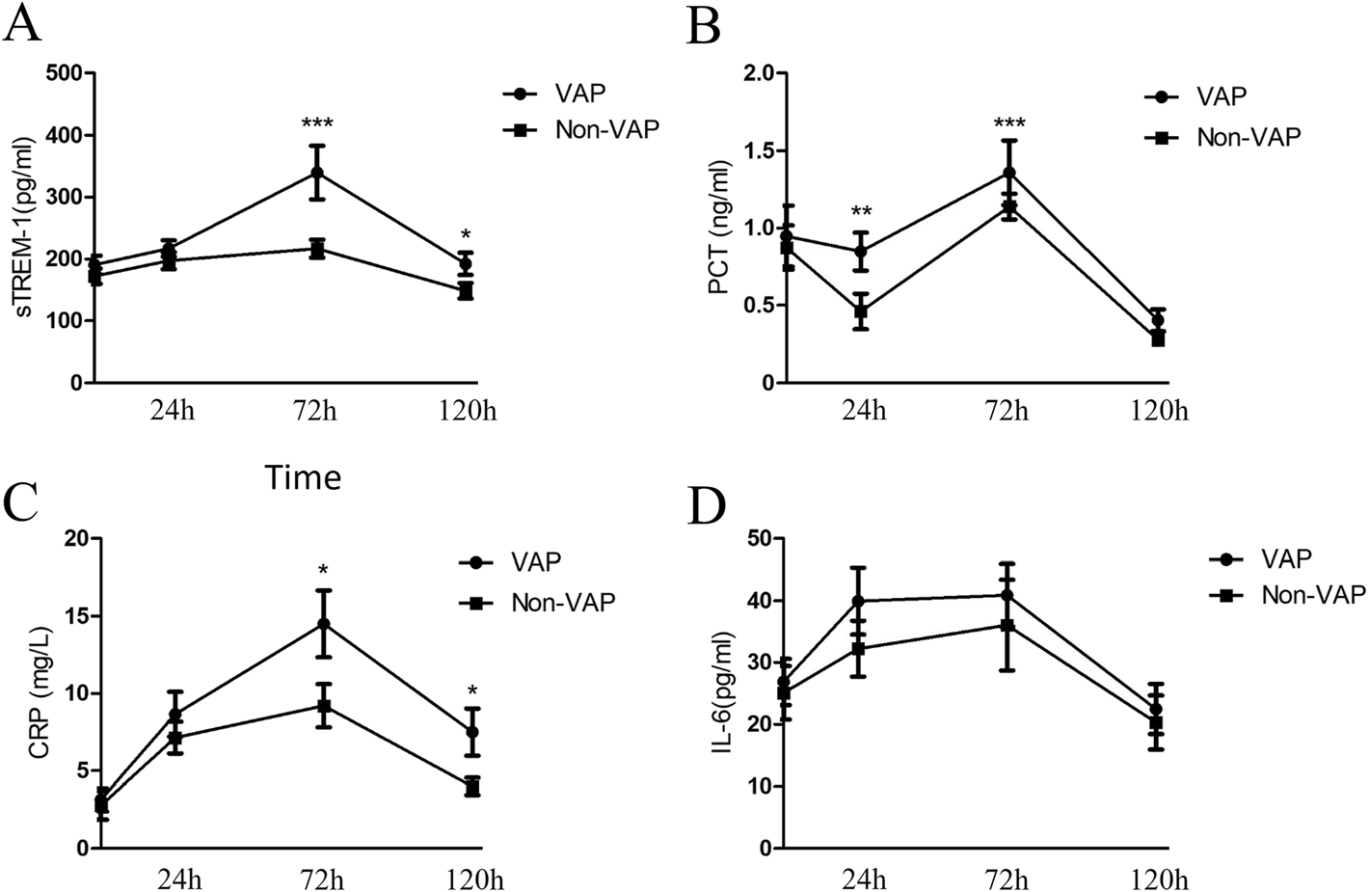
Serum concentrations of sTREM-1, PCT, CRP and IL-6 over 120 h of MV in the VAP and non-VAP groups. *P < 0.05 for VAP vs. non-VAP group

After initiation of MV, the serum sTREM-1 concentration was elevated, with peak values observed at 72 h and declining values observed thereafter in both groups (Fig. 1a). An increasing trend without statistical significance was noted from 0 and 24 h of MV in the VAP group and non-VAP group (*P* = 0.549 and *P* = 0.112, respectively). After 72 and 120 h of MV, the sTREM-1 levels in the VAP group were significantly increased to 289.5 (179.6–427.0) pg/ml and 183.9 (119.8–232.1) pg/ml, respectively, and these concentrations were significantly higher than those in the non-VAP group (*P* < 0.001 [< 0.001 after adjustment for age] and *P* = 0.042 [*P* = 0.040 after adjustment for age; Fig. 1a, Supplemental Table 1). Comparisons of sTREM-1 levels in different timepoints were displayed in Supplementary Figure 1, which showed an obvious increase of sTREM-1 in VAP group at 72 and 120 h.

### Dynamic changes in serum levels of inflammatory cytokines in patients with and without VAP

The serum PCT levels followed a trend similar to that of the serum sTREM-1 concentration during MV (Fig. 1b). The serum PCT level was significantly greater after 72 h of MV in patients who developed VAP than in those who did not [1.89 (1.38–2.61) vs 1.10 (0.75–1.36) pg/ml, *P* < 0.001]. The serum PCT level at 72 h in the VAP group was also significantly greater than those at 0, 24 and 120 h (all *P* < 0.001). The serum PCT levels after 24 and 120 h of MV appeared higher in the VAP group than in the non-VAP group, but the differences were not significant after adjustment for age. The serum CRP level after 72 h of MV was also significantly greater in the VAP group than in the non-VAP group (10.06(6.87–17.62) vs 7.97(4.43–12.56), *P* = 0.047 after adjustment for age; Fig. 1c). The serum IL-6 level did not differ between the two groups at any time point (Fig. 1d).

### Correlations between potential biomarkers and VAP

Potential correlations between the measured biomarkers and VAP were analyzed at 0, 24, 72 and 120 h of MV. A positive correlation was observed between the serum sTREM-1 concentration at 72 h and VAP (r = 0.697, *P* < 0.001). Positive correlations were also found between the serum PCT concentrations at 24 and 72 h and VAP (r = 0.429 and 0.601, respectively, both *P* < 0.05; Table 2).

**Table 2:** Correlations between potential biomarkers and VAP

Biomarkers	0 h		24 h		72 h		120 h	
	r	P	r	P	r	P	r	P
sTREM-1	0.079	0.549	0.208	0.111	0.697	< 0.001	0.266	0.040
PCT	−0.49	0.710	0.429	0.001	0.601	< 0.001	0.075	0.568
CRP	0.035	0.793	0.042	0.748	0.260	0.045	0.269	0.038
IL-6	0.166	0.204	0.135	0.305	0.204	0.118	0.162	0.217

### Predictive value of serum sTREM-1, PCT, CRP, and IL-6 for VAP based on logistic regression analysis

The serum concentrations of sTREM-1, PCT, CRP and IL-6 at baseline and after 120 h of MV were not associated with the diagnosis of VAP. However, the serum PCT concentration at 24 h was associated with VAP (odds ratio [OR] 2.724, 95% confidence interval [CI] 1.021–7.270, *P* = 0.045). At 72 h, the serum concentrations of both PCT (OR 50.309, 95%CI 4.045–625.720, *P* = 0.007) and sTREM-1 (OR 1.033, 95%CI 1.009–1.057, *P* = 0.007) were significantly associated with VAP. The Hosmer-Lemeshow test showed that the *P* value for the correlation between sTREM-1 and VAP was greatest at 72 h (*P* = 0.759 > 0.05), which implied a better degree of fitting.

### ROC curve analysis of the predictive value of serum sTREM-1, PCT, CRP and IL-6 for VAP

**Fig. 2:**
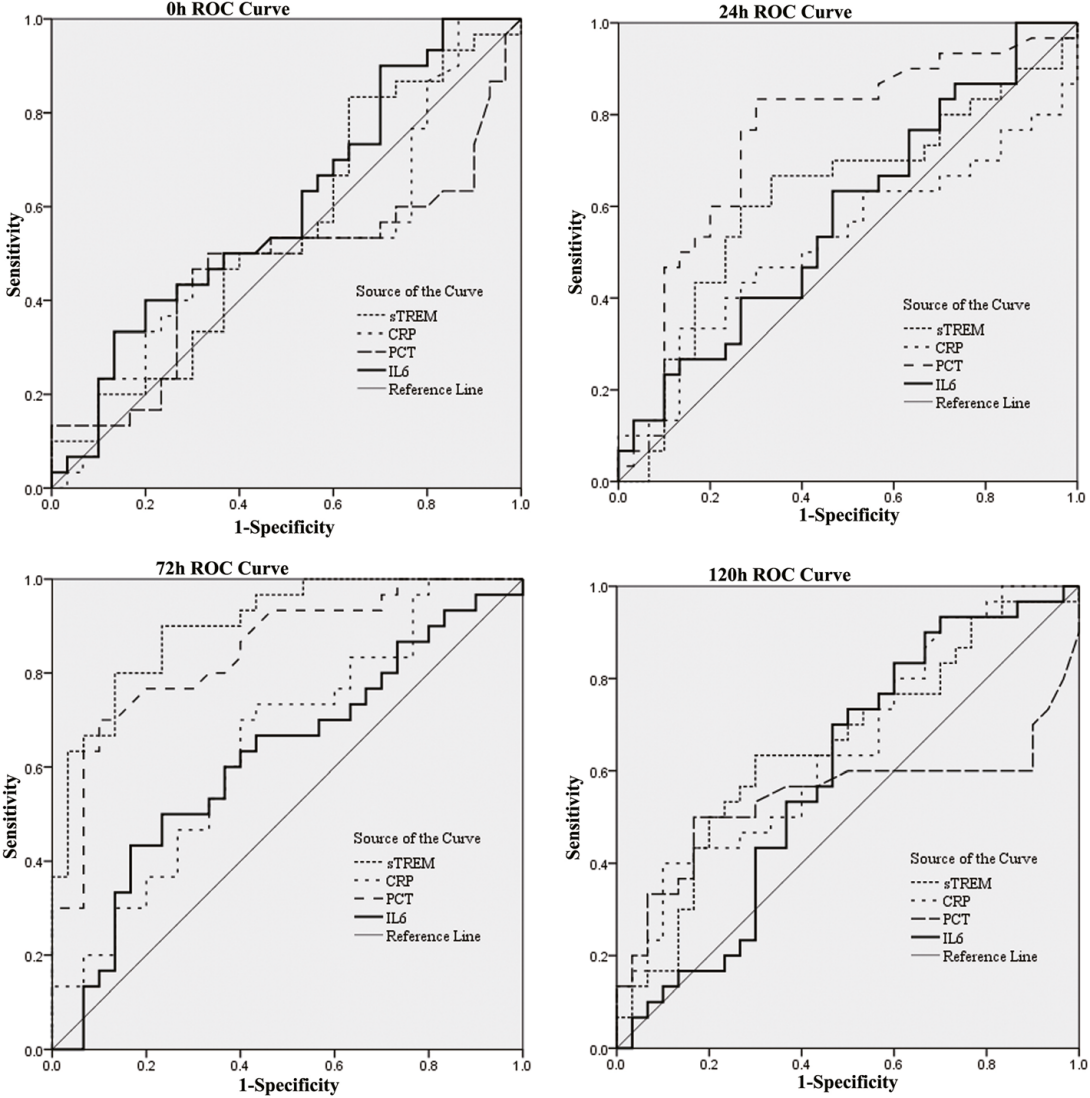
ROC curves for the ability of the serum sTREM-1, PCT, CRP and IL-6 concentrations at 0, 24, 72 and 120 h of MV to predict VAP

ROC curves for the ability of the serum sTREM-1, PCT, CRP and IL-6 at the four time points of MV to predict VAP were plotted (Fig. 2). The area under the curve (AUC) values were obviously greater after 72 h of MV, and at this time point, the optimal cutoff value for the serum sTREM-1 concentration was 165.05 pg/ml with an AUC of 0.902. The predictive sensitivity of sTREM-1 for VAP was 90% and the specificity was 77%. Meanwhile, the AUC values were smaller for the serum PCT, CRP and IL-6 concentrations (Fig. 2, Supplemental Table 2).

### ROC curves for the ability of a biomarker combination to predict VAP

**Fig. 3:**
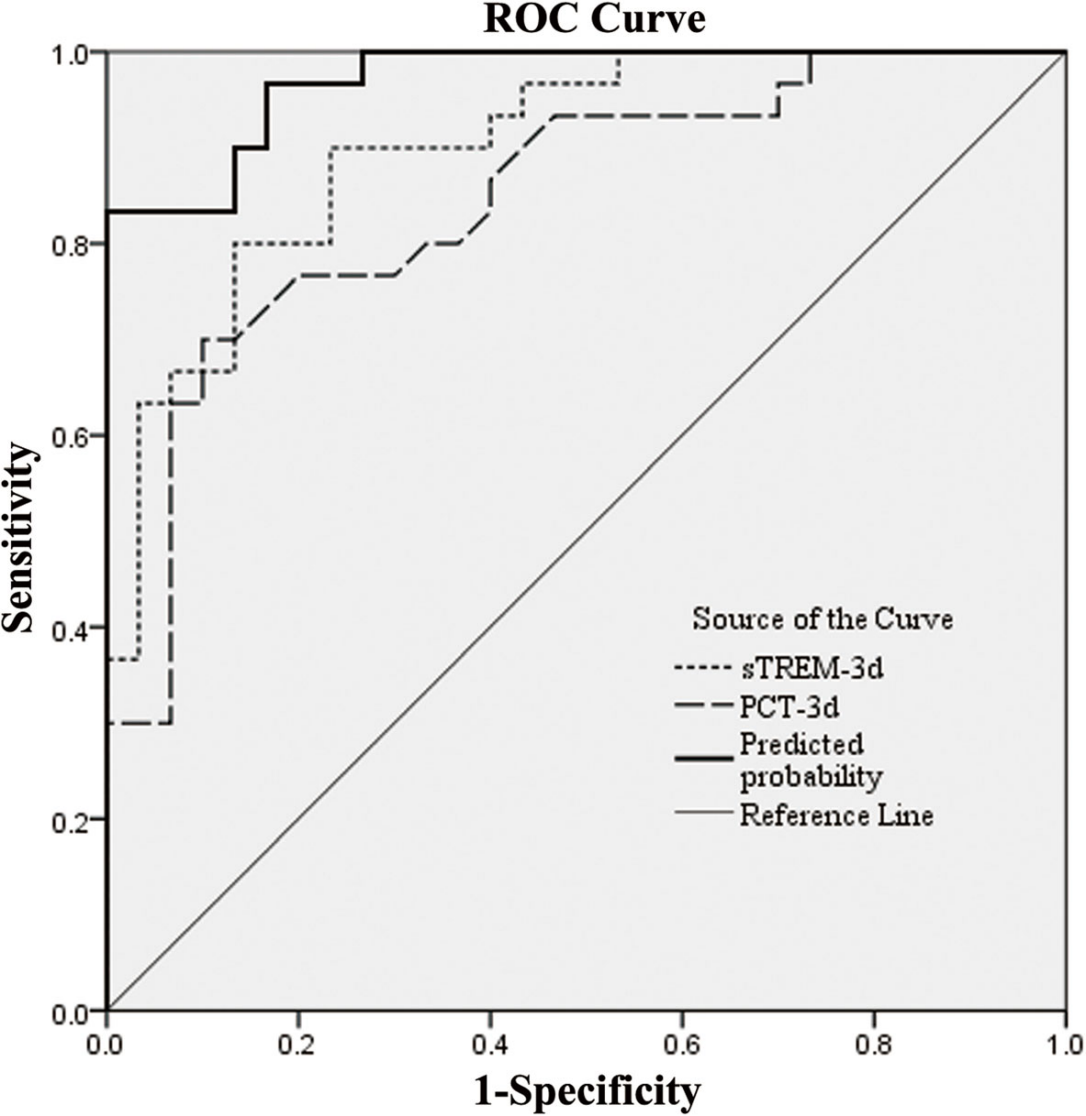
ROC curve for the ability of the combination of sTREM-1 and PCT to predict VAP

A ROC curve was constructed to examine the ability of the combination of serum sTREM-1 and PCT concentrations after 72 h of MV to predict VAP. The AUC, sensitivity, specificity and Youden index values were 0.971 (95% CI: 0.938–1.000), 0.96, 0.88 and 0.84, respectively, and these values were all better than those for either single marker. The corresponding prediction equation was as follows: *P* = 1/1 + e^-z^。Z = 0.027*sTREM-1 + 3.918*PCT, where P represents the predictive probability (0 ≤ *P* ≤ 1) and e is the natural logarithm (Fig. 3).

## Discussion

VAP is a frequently seen complication in neonates treated with MV, and it is associated with a high morbidity and mortality [2, 18]. Early recognition of VAP permits the timely implementations of treatment strategies [19], but unfortunately, early diagnosis of VAP is difficult mainly due to the lack of specific early clinical manifestations. The widely used approach of lower respiratory tract tissue culture is easily contaminated and may interfere with the diagnosis [20, 21]. The current study evaluated the predictive value of the serum concentration of sTREM-1, in addition to other conventional biomarkers including PCT, CRP and IL-6, in the early detection of VAP. Our findings revealed that the serum sTREM-1 concentration after 72 h of MV could well predict the occurrence of VAP in neonatal patients, especially when combined with the serum PCT concentration at 72 h of MV. Conversely, the serum concentrations of CRP and IL-6 did not exhibit solid predictive value for VAP in neonates.

sTREM-1 was reported to be a biomarker for the severity of infection [22]. In 2000, Bouchon A. et al. showed for the first time that TREM-1, as a member of the novel immunoglobulin super receptor family (Ig-superfamily, Ig-SF), triggers the release of chemokines and pro-inflammatory cytokines [23]. During acute infection, sTREM-1 is highly expressed and sheds from the cell surface into blood, saliva and other secretions. The dynamic blood level of sTREM-1 was shown to be associated with changes of disease severity [22]. In the current study, while the serum sTREM-1 concentrations were elevated in both groups of patients during the first 72 h of MV, patients in the VAP group had remarkably higher levels of sTREM-1 than those in the non-VAP group. MV alone can cause ventilator-associated lung injury (VALI) and enhance the release of multiple inflammatory cytokines. Therefore, it is not surprising that the sTREM-1 level was also increased in the non-VAP group with MV. However, for patients with VAP, systemic infection may contribute to the even higher levels of sTREM-1 that we observed. Gibot et al. found that the increased level of sTREM-1 in septic patients decreased as the condition improved [24]. In the current study, a similar pattern in the serum sTREM-1 concentration was observed.

Previous studies of the predictive value of sTREM in lung disease have reported controversial results. sTREM-1 was reported to be an independent predictive marker of pneumonia [24]. Another study evaluating the prognostic value of sTREM-1 in patients with lung diseases claimed that sTREM-1 is a powerful biomarker reflecting clinical outcome [25]. However, a conflicting result was reported by a previous study that assessed the predictive value of sTREM-1 in pediatric patients with VAP and found no difference in serum and bronchoalveolar lavage sTREM-1 concentrations between the VAP and non-VAP groups [26]. There are several possible reasons for the discrepant findings between this previous study and our present study. First, the present study focused on neonates, whereas most previous studies only enrolled pediatric or adult patients. In addition, the underlying diseases could be different in different study populations, which might affect the study results. Also, the serum sTREM-1 concentrations were measured by ELISA in the present study but determined by Western blotting in previous studies [27]. The use of different methods to measure sTREM-1 concentration might contribute to the disparity of the results. Moreover, different types of pathogens might affect the expression of sTREM-1. For example, bacterial and fungal infection can increase the expression of sTREM-1, whereas almost no change in the sTREM-1 level has been observed in cases of viral infection [28]. Finally, the sample size was rather limited in the present study, and further research with a larger sample size is necessary.

Combined measurement of sTREM-1 and PCT showed more robust predictive performance for VAP in neonates. The AUC, sensitivity, specificity and Youden index for the combination of these markers were all better than those for either single marker. In previous studies, combined measurement of serum PCT and BALF sTREM-1 concentrations was reported to have potential for the detection of nosocomial sepsis and for discriminating VAP versus extrapulmonary infection or infection versus non-infectious disease [29, 30]. These previous findings support our observation that the combined measurements are preferable to a single marker for the prediction of VAP.

The present study has several limitations. First, this was a pilot study, and the sample size was small. However, the significant value of sTREM-1 for VAP prediction was still obvious. Second, all patients received antibiotics, and the effects of antibiotic treatment on the measurement results were not studied. Third, healthy neonates who did not require MV were not included in the study as a control group. Fourth, as we noticed, non-VAP group also had an increased level of sTREM-1 possibly caused by VALI. However, we did not perform bacterial or fungal cultures in non-VAP patients due to the study design. For adults, a previous study found that sTREM-1 was present in a high concentration in the BALF of patients with bacterial VAP. However, the relationship between sTREM-1 level and microbial growth was not evaluated [31]. For neonates, obtaining a BALF specimen without contamination is often clinically infeasible partly because the neonate’s airway is delicate. In addition, it’s difficult to distinguish colonization from infection [32]. Fifth, the cut-off level of many inflammatory biomarkers may be depended on postnatal age. In addition, different measurement may also affect the cut-off level. However, due to the limited sample size, we did not further analyze the sTERM-1 level in patients with different postnatal age. Further large-scale studies might provide more information on the features of sTREM-1 in neonatal VAP.

In conclusion, the serum sTREM-1 concentration was found to be a reliable biomarker for the prediction of VAP in neonatal patients treated with MV, and the combination of sTREM-1 and PCT concentrations showed even greater predictive power for VAP in these patients. Further large-scale multicenter trial is suggested to confirm our findings.

## Supplementary information

**Additional file 1: Supplemental Table 1.** Serum sTREM-1 concentrations in neonatal patients in the VAP and non-VAP groups. MV: Mechanic ventilation; ^*^P after adjustment for age

**Additional file 2: Supplemental Table 2.** Predictive performance of serum sTREM-1, PCT, CRP and IL-6 concentrations at 0, 24, 72 and 120 h of MV AUC: area under the curve; CI: confidence interval; PPV: positive predictive value; NPV: negative predictive value; PPR: positive probability ratio; NPR: negative probability ratio

**Additional file 3: Supplementary Figure 1.** Comparison of sTREM-1 levels at 0, 24, 72 and 120 h in VAP and non-VAP groups. ^*^P<0.05, ^***^P<0.001

## Data Availability

The data and materials supporting the conclusions of the study are available from the corresponding author on reasonable request.

## Abbreviations

AUC: Area under the curve
BALF: Bronchoalveolar lavage fluid
CRP: C-reactive protein
CDC: Centers for Disease Control and Prevention
ELISA: Enzyme-linked immunosorbent assay
ICU: Intensive care unit
IL-6: Interleukin-6
sTREM-1: Myeloid cells-1
MV: Mechanical ventilation
NICU: Neonatal Intensive Care Unit
NHSN: National Hospital Safety Monitoring Network
OR: Odds ratio
PCT: Procalcitonin
ROC: Receiver operating characteristic
SD: Standard deviation
VAP: Ventilator-associated pneumonia
VALI: Ventilator-associated lung injury
