# Supplementary material for: Significance of sTREM-1 in early prediction of ventilator-associated pneumonia in neonates: a single-center, prospective, observational study
Source: BMC Infect Dis. 2020 Jul 25;20:542. doi: 10.1186/s12879-020-05196-z (PMC7381866; doi:10.1186/s12879-020-05196-z)
Supplement: Supplementary file 1 — Additional file 1: Supplemental Table 1. Serum sTREM-1 concentrations in neonatal patients in the VAP and non-VAP groups. MV: Mechanic ventilation; *P after adjustment for age [file 12879_2020_5196_MOESM1_ESM.docx]

**Supplemental Table 1.** Serum sTREM-1 concentrations in neonatal patients in the VAP and non-VAP groups.

| Time point | VAP group (n=30) | Non-VAP group (n=30) | P |
| --- | --- | --- | --- |
| MV 0 h | 180.5(145.5-210.4) | 178.4(110.7-215.0) | 0.277 |
| MV 24 h | 214.7(159.0-287.6) | 183.9(152.4-211.3) | 0.112 |
| MV 72 h | 289.5(179.6-427.0) | 202.9(154.8-279.6) | <0.001^*^ |
| MV 120 h | 183.9(119.8-232.1) | 141.3(99.8-179.1) | 0.042^*^ |

MV: Mechanic ventilation; ^*^P after adjustment for age
