# Supplementary material for: Significance of sTREM-1 in early prediction of ventilator-associated pneumonia in neonates: a single-center, prospective, observational study
Source: BMC Infect Dis. 2020 Jul 25;20:542. doi: 10.1186/s12879-020-05196-z (PMC7381866; doi:10.1186/s12879-020-05196-z)
Supplement: Supplementary file 2 — Additional file 2: Supplemental Table 2. Predictive performance of serum sTREM-1, PCT, CRP and IL-6 concentrations at 0, 24, 72 and 120 h of MV AUC: area under the curve; CI: confidence interval; PPV: positive predictive value; NPV: negative predictive value; PPR: positive probability ratio; NPR: negative probability ratio [file 12879_2020_5196_MOESM2_ESM.docx]

**Supplemental Table 2.** Predictive performance of serum sTREM-1, PCT, CRP and IL-6 concentrations at 0, 24, 72 and 120 h of MV

| Variable | sTREM-1 (pg/ml) | PCT (ng/ml) | CRP (mg/l) | IL-6 (pg/ml) |
| --- | --- | --- | --- | --- |
| **0 hours** |  |  |  |  |
| AUC | 0.546 | 0.528 | 0.52 | 0.59 |
| Cut-off value | 125.65 | 1.14 | 8.45 | 34.91 |
| P | 0.549 | 0.718 | 0.796 | 0.196 |
| 95% CI | 0.412 -0.675 | 0.395-0.659 | 0.387-0.651 | 0.462-0.721 |
| Sensitivity | 0.83 | 0.37 | 0.57 | 0.4 |
| Specificity | 0.37 | 0.9 | 0.23 | 0.8 |
| PPV | 56.8 | 78.6 | 42.5 | 66.7 |
| NPV | 68.7 | 58.7 | 35.0 | 57.5 |
| PPR | 1.32 | 3.67 | 0.74 | 2.0 |
| NPR | 0.45 | 0.7 | 1.86 | 0.75 |
| Youden index | 0.2 | 0.267 | 0.2 | 0.2 |
| **24 hours** |  |  |  |  |
| AUC | 0.62 | 0. 748 | 0.524 | 0.578 |
| Cut-off value | 197.65 | 0.35 | 9.56 | 22.5 |
| P | 0.113 | 0.000 | 0.753 | 0.298 |
| 95% CI | 0.485 -0.742 | 0.624-0.852 | 0.391-0.655 | 0.443-0.704 |
| Sensitivity | 0.67 | 0.83 | 0.33 | 0.63 |
| Specificity | 0.67 | 0.70 | 0.87 | 0.53 |
| PPV | 66.7 | 73.5 | 71.4 | 57.6 |
| NPV | 66.7 | 80.8 | 56.5 | 59.3 |
| PPR | 2 | 2.78 | 2.5 | 1.36 |
| NPR | 0.5 | 0.24 | 0.77 | 0.69 |
| Youden index | 0.33 | 0.53 | 0.2 | 0.17 |
| **72 hours** |  |  |  |  |
| AUC | 0.902 | 0.847 | 0.650 | 0.618 |
| Cut-off value | 165.05 | 1.55 | 8.43 | 39.91 |
| P | 0.000 | 0.000 | 0.046 | 0.117 |
| 95% CI | 0.828-0.976 | 0.748-0.945 | 0.511-0.789 | 0.474-0.762 |
| Sensitivity | 0.9 | 0.67 | 0.73 | 0.5 |
| Specificity | 0.77 | 0.87 | 0.57 | 0.77 |
| PPV | 79.4 | 87.5 | 61.8 | 68.2 |
| NPV | 88.5 | 75 | 65.4 | 60.5 |
| PPR | 3.86 | 7 | 1.62 | 2.14 |
| NPR | 0.13 | 0.33 | 0.53 | 0.65 |
| Youden index | 0.67 | 0.54 | 0.3 | 0.27 |
| **120 hours** |  |  |  |  |
| AUC | 0.653 | 0.543 | 0.655 | 0.593 |
| Cut-off value | 165.21 | 0.33 | 8.10 | 9.91 |
| P | 0.034 | 0.599 | 0.029 | 0.217 |
| 95% CI | 0.519-0.772 | 0.410-0.673 | 0.521-0.773 | 0.459-0.718 |
| Sensitivity | 0.63 | 0.50 | 0.35 | 0.83 |
| Specificity | 0.70 | 0.83 | 0.90 | 0.40 |
| PPV | 67.9 | 70.6 | 77.2 | 58.1 |
| NPV | 65.6 | 58.1 | 58.4 | 70.6 |
| PPR | 2.11 | 2.4 | 3.62 | 1.39 |
| NPR | 0.52 | 0.72 | 0.73 | 0.42 |
| Youden index | 0.34 | 0.33 | 0.3 | 0.23 |

AUC: area under the curve; CI: confidence interval; PPV: positive predictive value; NPV: negative predictive value; PPR: positive probability ratio; NPR: negative probability ratio
