# Supplementary figures and images for: Significance of sTREM-1 in early prediction of ventilator-associated pneumonia in neonates: a single-center, prospective, observational study
Source: BMC Infect Dis. 2020 Jul 25;20:542. doi: 10.1186/s12879-020-05196-z (PMC7381866; doi:10.1186/s12879-020-05196-z)

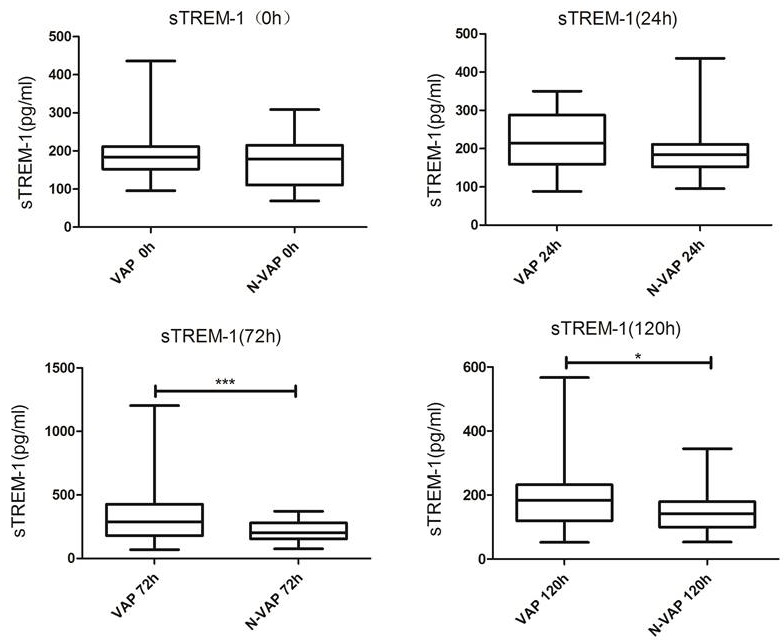

Supplement: Supplementary file 3 — Additional file 3: Supplementary Figure 1. Comparison of sTREM-1 levels at 0, 24, 72 and 120 h in VAP and non-VAP groups. *P<0.05, ***P<0.001 [file 12879_2020_5196_MOESM3_ESM.jpg]
